# Informed Strategies Based on Education Research to Enhance the Learning Ecosystem

**DOI:** 10.7759/cureus.69431

**Published:** 2024-09-14

**Authors:** Mohammad I Qureshi, Tripti Shrivastava, H V Sharath, Gurjeet Kaur

**Affiliations:** 1 Department of Neurophysiotherapy, Ravi Nair Physiotherapy College, Datta Meghe Institute of Higher Education and Research (Deemed to be University), Wardha, IND; 2 Department of Physiology, Jawaharlal Nehru Medical College, Datta Meghe Institute of Higher Education and Research (Deemed to be University), Wardha, IND; 3 Department of Paediatric Physiotherapy, Ravi Nair Physiotherapy College, Datta Meghe Institute of Higher Education and Research (Deemed to be University), Wardha, IND; 4 Department of Physiotherapy, Center for Advance Physiotherapy Education (CAPER) Ravi Nair Physiotherapy College, Datta Meghe Institute of Higher Education and Research (Deemed to be University), Wardha, IND

**Keywords:** flipped classroom, health professional's education, problem-based learning (pbl), self-directed learning (sdl), student education

## Abstract

Educational institutions must change to create a learning ecosystem that prioritizes skills crucial for the 21st-century learners in the current global context that is quickly evolving. With an emphasis on evidence-based education, global citizenship, the integration of Sustainable Development Goals (SDGs), translatory practice, active learning strategies, improved clinical placement experiences, and peer learning, this paper explores the strategies for enhancing the efficacy of learning ecosystems. These elements are essential for encouraging continuous education in students and preparing them to meet their personal and professional needs. Through the integration of these components into instructional strategies, teachers can cultivate an atmosphere that readies students for the intricacies of contemporary work environments and global citizenship.

## Introduction and background

The idea of a learning ecosystem exceeds conventional classroom instruction, incorporating various stakeholders, resources, and approaches to promote holistic growth in students. As technology and globalization change the face of education, creating comprehensive learning environments that equip students to tackle global issues is becoming increasingly important. Evidence-based teaching strategies, the creation of global citizenship competencies, alignment with the Sustainable Development Goals (SDGs), and the encouragement of translation practices, which make research understandable and useful in practical settings, are essential components of this strategy. A complete educational experience also requires improving clinical placement experiences, promoting peer learning opportunities, and supporting active learning [1].

Education is changing quickly in response to cultural shifts, technology breakthroughs, and demands from throughout the world. In order to develop lifelong learners who are prepared to handle the complexity of the twenty-first century, modern learning environments must change. A learning ecosystem is a dynamic, all-encompassing setting where different components work together to promote and improve learning. It combines academic education with real-world applications, digital tools, and cooperative learning techniques. It also blends formal and informal learning possibilities [2,3].

This study investigates how educational institutions might create learning ecosystems that tackle the opportunities and problems of the modern world. Evidence-based instruction, global citizenship, incorporating the SDGs, translational methods, active learning, improved clinical placement experiences, and peer learning are the main areas of emphasis. These elements are crucial for equipping students to succeed academically as well as to actively engage in a worldwide society.

## Review

Methodology

The study uses a mixed-methods approach, including case studies, qualitative insights from educators, and a survey of recent research. There are three primary stages in the research methodology used. Literature review is the initial phase encompassing a thorough analysis of books, papers, and peer-reviewed articles that have been gathered from scholarly databases including Google Scholar, Scopus, PubMed, and Education Resources Information Center (ERIC). The main topics of the literature study include active learning, global citizenship, evidence-based education, translation practices, SDGs, and clinical placements. Research in these domains that showcase novel approaches, cutting-edge tactics, and practical implementations receive particular emphasis. The review also points out gaps in the body of knowledge, which help explain the suggestions made in the sections that follow. 

Analyzing case studies from educational institutions that have effectively included components of a successful learning ecosystem is part of the second step. These case studies are taken from a variety of settings, such as global citizenship initiatives, health professions education, and postsecondary educational establishments. The study offers useful insights into how the suggested tactics might be implemented in various educational contexts by looking at real-world instances.

Qualitative synthesis is the third step involves compiling the qualitative information gathered from surveys and interviews with academic leaders, educators, and curriculum designers. The main objective is to acquire knowledge about the potential and difficulties involved in developing learning environments that support modern educational objectives. The conclusions drawn from the case studies and literature analysis are validated and supported by the qualitative data.

Review

Translation of Educational Research in Evidence-based Education

Making judgments about education and putting plans into action based on facts and empirical study is known as evidence-based education. Better learning results are the outcome of the use of evidence-based techniques, which guarantee that treatments and teaching strategies are based on tried-and-true effectiveness. It has been demonstrated, for example, that incorporating spaced repetition and active recall studies from cognitive science into curriculum design improves student retention and mastering of challenging material.

Soicher et al. (2020) combined information from educational psychology, cognitive psychology, the science of learning, and discipline-based educational research to identify successful teaching and learning practices in higher education. To close the gap between research labs and college classrooms, they underlined the need for fruitful collaboration between researchers and instructors. They did point out that this translation gap has not been sufficiently reduced by translational and practice-based research on its own. They offer a unique model that takes implementation science into account in order to solve this problem. This model systematically examines the unpredictability and variability of implementing cognitive science treatments in actual educational environments. This approach seeks to provide cognitive scientists with a better understanding of the variables affecting how their research gets adopted in educational contexts. Teachers need useful information from researchers in order to enhance teaching and learning. There are four specific ways that cognitive researchers can work with implementation science: they should adopt planning and evaluation frameworks, think of teachers as scientist-educators, use pragmatic controlled trials in research designs, and increase reporting transparency. Through the application of these strategies, researchers and educators may work together to transform promising principles of cognitive science into practical classroom practices, which will ultimately support the integration of research findings into higher education policy and practice [2].

In order to successfully apply evidence-based education in classrooms with co-teachers, Wexler et al. (2021) suggest a methodical approach that is summed up in the acronym FIRST. This approach comprises two crucial planning recommendations.

1. Monitor fidelity: Co-teachers should create a plan to evaluate how accurately they apply evidence-based methods. Better student results are associated with high fidelity of implementation, which includes both adherence to instructional practices and the caliber of delivery. Checklists are one tool that may be used to monitor adherence and pinpoint areas that need work.

2. Combine methods: Throughout the academic year, evidence-based approaches should be smoothly integrated into everyday instruction to give students regular chances to interact with texts while receiving literacy training [3].

Global Citizenship Education

The goals of global citizenship education (GCE) are to increase students' social responsibility, cross-cultural competency, and awareness of global challenges. Teachers can encourage children to think critically about global issues and take local actions that lead to sustainable solutions by including GCE into their curricula. International collaborations, study abroad initiatives, and projects are all powerful tools for advancing global citizenship. Students are better prepared to discuss difficult global topics like social justice, climate change, and inequality when global citizenship education is included into the learning ecosystem. The goal of GCE is to promote tolerance, understanding, and a sense of collective responsibility for addressing global issues (UNESCO, 2015). Students can significantly impact a linked world by learning to understand other cultures and ideas through GCE [4].

Integration of Sustainable Development Goals (SDGs)

The SDGs of the United Nations offer a roadmap for tackling urgent global issues like poverty and climate change. The SDGs are incorporated into the curriculum to guarantee that students understand them and are able to contribute to them by coming up with creative solutions [5]. By coordinating course material with pertinent SDG objectives and supporting interdisciplinary approaches that integrate theory and practice, educational institutions can support this endeavor.

The SDGs must be integrated into higher education. This can be done in a number of ways, including through knowledge, learning, teaching, institutions, methodology, competence, and course design. In addition to programs like ecological, change agency, and infrastructural initiatives, behavioral settings, subjects, and curricula are examples of knowledge production domains for change. Public debate integration involves audience and impact considerations. Providing public services and advocacy are also important dimensions [5,6]. This concept can enhance graduates' understanding of SDGs and increase public awareness through service provision and implementation in higher education institutions. Aligning the National Education Policy (NEP) 2020 with SDGs can greatly benefit higher education institutions by promoting quality education and sustainable development, as they share common objectives related to access, learning outcomes, innovation, research, and teacher training. Students prefer to adopt SDGs, such as decent work, quality education, industry, innovation, infrastructure, and sustainable cities, as their future career choices. Suggestions for improving Education for Sustainable Development (ESD) implementation include comprehensive formal and non-formal education, publicity, and curriculum integration to promote learning about SDGs and enhance students' awareness, knowledge, competencies, and engagement in global sustainable development [7]. 

Translatory Practices

Translatory practice refers to the application of theoretical knowledge in real-world contexts. By incorporating project-based learning, internships, and community engagement initiatives, educators can help students bridge the gap between academic learning and practical application. Translatory practices are essential in disciplines such as healthcare, engineering, and social sciences, where theoretical knowledge needs to be translated into actionable skills [8].

Translator practices aim to close the gap between research and practice by providing practitioners and policymakers with accessible and relevant research results. These procedures aid in the translation of scholarly research into forms that have an immediate influence on educational policies and initiatives. For instance, employing meta-analyses and systematic reviews to direct curriculum creation guarantees that instructional strategies are consistently improved in light of the most recent research [9,10].

In translation studies, Schön et al. (2017) highlight a thorough teaching methodology that supports an integrated approach that combines academic knowledge with practical abilities. This approach uses a well-organized design that combines several instructional techniques, such as seminars, workshops, and group projects. In order to help students grasp the complexity inherent in translation, interactive sessions involving real-world translation problems are crucial. A reassessment of translator education that strikes a balance between theoretical and practical aspects is necessary because translation studies is a dynamic area that is always evolving as a result of globalization, technological improvements, and altering cultural contexts. In order to educate students for contemporary translation processes, educators are urged to use multidisciplinary techniques that draw from linguistics, cultural studies, and technology [9].

In order to improve efficiency and accuracy, students must learn how to traverse a variety of tools and resources, which is where the translator's workstation comes into play. In order to promote translator competence, assessment in translator education should take a multidimensional approach, assessing students' abilities to apply translation procedures, analyze texts, and reflect on their work. Last but not least, the transition in second language learning from traditional methods to more holistic approaches emphasizes the significance of context and interaction in language learning, readying students for the challenges of translation where cultural nuances and contextual understanding are critical [10,11].

Active Learning

Compared to typical lectures, active learning methodologies like problem-based learning (PBL), flipped classrooms, and peer instruction engage students more profoundly. Active learning enhances recall, critical thinking, and problem-solving abilities, according to research. A learning environment that is more engaging and participative can be created by switching from teacher-centered to student-centered education. Active learning techniques place a strong emphasis on student participation and engagement through group projects, case studies, discussions, and problem-solving. Studies have consistently demonstrated that students who participate in active learning attain superior results when compared to pupils in conventional lecture-based environments. Using strategies like think-pair-share, peer education, and flipped classrooms improves retention and fosters critical thinking [12].

Moreover, interactive learning strategies, such as small groups, team-based learning, discussions, cooperative learning, games, role-playing, questioning, and debates, alongside critical thinking strategies like problem-based learning, case-based learning, simulations, project-based learning, and inquiry-based learning, were examined. Through the open coding process, various themes emerged: first, active learning is rooted in student-centered constructivist theory, indicating a specific pedagogical orientation; second, active learning serves to foster higher-order thinking and deep learning, which points to the expected outcomes; and third, active learning is characterized by participation and engagement, reflecting the more prevalent expressions of this educational approach [13].

Enhancing Clinical Placement Experiences

Clinical placements and real-world experience are essential for careers in disciplines like teaching, engineering, and healthcare. Students' professional abilities and confidence can be significantly increased by structured placements that incorporate clear learning objectives, reflective practice, and mentorship opportunities. To provide meaningful and pertinent placement experiences, educational institutions and business must work together in collaborative relationships [14]. Clinical rotations are an essential part of the curriculum for students pursuing careers in the health and medical industries. Successful clinical placements are an essential part of their training. Good clinical internships give students practical experience and enable them to put their academic knowledge to use. Techniques including feedback loops, mentorship programs, and reflective practice can improve learning during placements, resulting in improved confidence and skill gain [15].

Students who took part in interprofessional student-run clinics (SRCs) gained a deeper comprehension and respect for comprehensive and team-based patient care, according to Yap et al. (2024). Students had a deeper understanding of the scope of practice, the roles that they and others play, and the value of making a meaningful contribution to a team by working with practitioners from a variety of health professions and managing actual patients. They felt more at ease working in interprofessional teams as a result of this experience, which gave them excellent chances to cooperate and forge interprofessional bonds with clients and coworkers. Students were able to obtain real-world experience and hone their skills in a community-partnered primary healthcare setting by collaborating with community health providers [16].

Research and policy in teacher education advocate for special education (SPED) programs to provide candidates with supervised field experience opportunities that serve several purposes are (a) immerse candidates in professional practice, (b) alleviate their anxiety, (c) help them build confidence, and (d) enable them to see themselves as educators [17].

Encouraging Peer Learning

Peer learning leverages students’ ability to teach and learn from each other. Collaborative activites such as group projects, peer assessment, and study groups not only enhance understanding but also build teamwork, communication, leadership skills. Peer tutoring, study groups, and collaborative projects provide opportunities for deeper understanding and collective knowledge construction [18]. As a result, they recommend increasing the use of collaborative learning activities in the classroom to enhance peer learning opportunities. Research suggests that peer earning can significantly improve academic performance, especially when combined with active learning techniques [18]. Peer learning involves students learning from and with each other, leveraging collaboration and social interaction as key elements of the educational process. This method has proven effective in developing communication, leadership, problem-solving skills [19].

According to Ipek et al. (2021), blended learning is a method that combines face-to-face instruction with online learning environments [20]. Modern learners are expected to possess problem-solving abilities, collaborate effectively, and communicate well. It is believed that peer learning can enhance these skills within constructivist teaching frameworks. Teacher candidates generally express positive views on peer learning conducted through interactive videos that include questions during the blended learning process. Additionally, even in online learning environments, digital assessment has a good impact on group dynamics and peer learning [21].

The zone of proximal development, core scaffolding principles, and scaffolding tactics based on sociocultural theory are all incorporated into a conceptual model for peer learning in higher education settings. The methodology consists of four steps: (a) introductions; (b) group education; (c) evaluation of oneself; and (d) completing the peer learning process. It also takes into account how scaffolding affects the structure of peer learning activities and offers a framework within which peers can choose the most suitable scaffolding strategies. By combining the theoretical underpinnings, conceptualization, and operationalization of scaffolding strategies, this approach seeks to improve the efficacy of peer learning in higher education [22], which was supported by Woodward and Pattinson (2023) who found that the most effective peer learning experiences occur during in-class activities combined with group assessments [23].

Use of Technology

The way that education is delivered has changed as a result of technology, making learning more flexible and personalized. Access, engagement, and learning outcomes are improved by the combination of learning management systems (LMS), virtual classrooms, and AI-driven adaptive learning platforms. Teaching tactics might be further optimized by utilizing data analytics from these platforms [24,25].

In order to improve accessibility and innovation within digital learning ecosystems, Rojas and Chiappe (2024) explored the possibilities of a number of technologies, including mobile devices, big data, augmented reality, learning analytics, natural interaction technologies, and semantic technologies. They emphasized the use of blockchain technology in conjunction with learning analytics to track students' development [26]. Furthermore, the goal of the integration of gamification and massive open online courses (MOOCs) is to produce more dynamic and captivating learning environments. Artificial intelligence (AI) is particularly well-known for its capacity to spot trends in student performance, pointing out areas that might not have received enough educational attention and enabling the development of stronger educational infrastructure and programs. AI also improves educational services with chatbots and virtual assistants, which can evaluate large amounts of data and offer real-time assistance, individualized learning recommendations, real-time support, and tailored feedback. 

In order to effectively teach children with special needs through hybrid learning models, teachers must be able to adapt to the quickly emerging technology and incorporate them into the learning process. In order to help students understand the information, this strategy necessitates using a variety of digital platforms as learning delivery technologies, including Google Classroom, WhatsApp, video conferencing, and other applications. Instructors stress how important it is to comprehend technology advancements, especially when it comes to boosting student-teacher relationships and improving the educational process. In the contemporary educational landscape, the mix of technology-enabled online learning and in-person instruction is viewed as critical to meeting students' learning demands [27]. 

Emphasizing Critical Thinking and Problem-solving Skills

Students need to develop critical thinking and problem-solving skills in order to flourish in a world that is complex and changing quickly. The goal of educational practices should be to promote information synthesis, analysis, and inquiry. Students can tackle obstacles with creativity and resilience when they participate in inquiry-based learning (IBL) and problem-based learning (PBL) programs, which are especially successful at fostering these skills [28].

The application of a problem-based learning (PBL) model with five essential steps was examined by Susanti al. (2023). These steps are: introducing students to the problem; setting them up for study; supporting individual and group investigations; assisting in the development and presentation of solutions; and assessing and evaluating the problem-solving process. According to the study, instructors and students thought that problem-solving and critical thinking skills may be improved with the use of PBL [29]. The results, which are corroborated by Maksum et al. (2021), show that social skills, problem-solving abilities, self-control, and critical thinking abilities positively influence learning outcomes [30].

Wang et al. (2023) underscore the importance of critical thinking and problem-solving skills (CPS) as emphasized by the Organization for Economic Co-operation and Development (OECD) in the 21st century. These skills are linked to enhanced academic performance, career development, and job competency training. Reflective learning, which includes journal writing, peer reflection, self-reflection, and group discussions, has been identified as a method to strengthen critical thinking and problem-solving abilities. This process of learning through experience is essential in professional education, aiming to enhance various skills such as insight, empathic concern, computational thinking, and problem-solving. Factors influencing CPS skills include algorithmic thinking, cooperativity, creativity, and empathic concern, with studies showing that both empathic concern and critical thinking significantly impact problem-solving abilities. The researchers proposed a theoretical model that identifies algorithmic thinking, creativity, and cooperativity as antecedents, with critical thinking acting as an intermediary variable affecting CPS skills. Additionally, personal distress, perspective-taking, and fantasy were considered as antecedents, with empathetic concern also serving as an intermediary variable. The findings indicate that reflective learning, grounded in multidimensional empathy theory and 21st-century skills theory, can significantly enhance CPS skills among medical students. These results suggest that educators should integrate reflective learning strategies that emphasize empathy and critical thinking into their curricula to improve CPS skills effectively [31].

The scoping review by Ghani et al. (2021) identifies key elements of effective learning behaviors within a problem-based learning (PBL) framework, categorized into three main areas. First, intrinsic empowerment encompasses proactivity, where students actively analyze problems, identify learning needs, seek guidance, and integrate knowledge across disciplines. Organization is also crucial, as effective learners set goals, plan, and monitor their progress. Diligence, characterized by persistence and hard work, is essential for success in PBL, along with resourcefulness in finding and utilizing appropriate learning materials. Second, under entrustment, students engage in self-assessment and peer assessment, which fosters critical thinking and self-directed learning. Taking on teaching roles reinforces their understanding, while providing and receiving constructive feedback enhances their learning experience. Third, functional skills include time management, digital proficiency, data management, and collaboration. Effective time management is vital for balancing group work and self-directed learning, while digital proficiency improves learning efficiency. Organizing and managing information is key to effective problem-solving, and strong collaboration skills, such as communication and teamwork, are critical for success in PBL. By nurturing these effective learning behaviors, educators can equip students with the skills necessary for success in PBL and lifelong learning [32]. 

According to McLaughlin et al. (2019), design thinking is an innovative approach in health professions education that provides numerous advantages for teaching and learning. This method has been utilized as both a subject and a tool in the education and training of practitioners, patients, and students. Participants engaged in the design thinking process, focusing on the initial stages of inspiration and ideation through activities such as lectures, small group discussions, and workshops. The findings indicated improvements in self-efficacy, participant experiences, program characteristics, and solutions to specific challenges. Key benefits of design thinking include fostering collaboration among multidisciplinary teams and diverse stakeholders, enhancing interdisciplinary communication skills, refining problem identification with a human-centered approach, and advancing creativity and communication skills that ultimately improve patient outcomes. Additionally, design thinking acknowledges the significance of context and its adaptability to various time scales, industries, and situations. In summary, design thinking presents a promising strategy for health professions education, encouraging creativity, collaboration, and innovation in tackling complex healthcare issues [33].

Promoting Ethical and Reflective Practice

Reflective practice involves continuous self-evaluation and reflective on learning experiences, which is crucial for lifelong learning. By incorporating reflective exercises such as journals, portfolios, and critical incident analysis into the curriculum, educators can help student develop self-awareness and critical thinking skills necessary for continuous personal and professional development. Ethical and reflective practice is vital for developing professionals who are not only competent but also socially responsible. Encouraging students to reflect on their learning experiences, consider ethical dilemmas, and apply moral reasoning helps build integrity and accountability in their professional lives [34].

Reflection offers significant benefits, including enhanced confidence, organization, time management, prioritization, communication, and teamwork. It is a lifelong skill that can be applied academically, personally, and professionally. Importantly, when students engage in reflection, it can profoundly impact their career paths and choices. Therefore, it is essential to introduce reflective practices early, even at the primary school level, to foster a lifelong journey of reflection that enhances students' thought processes and aids in decision-making and course selection [35].

Reflective practice is closely linked to the professional growth and development of preservice teachers, as it enables them to learn from their experiences, construct new knowledge, and apply it to future situations. Through reflection, preservice teachers can improve their classroom management skills, establish effective classroom norms and policies, assess their teaching based on feedback from students, mentor teachers, and professors, and enhance their lesson planning and student interaction abilities. Reflective practice also equips preservice teachers with strategies to deal with challenges that arise in the classroom and build positive relationships with students and colleagues. Additionally, reflection helps preservice teachers identify their strengths and weaknesses, leading to increased self-confidence, self-assessment, acquisition of new knowledge and skills, and enthusiasm for the teaching profession. In essence, reflective practice is a crucial component of the education process, as it facilitates the development of effective and confident preservice teachers [36,37].

Morris-Eyton and Pretorius E (2023) explore the use of a digital promise alongside innovative virtual teaching methods to assess its potential in motivating student learning and enhancing accountability. Their findings suggest that fostering awareness of personal responsibility for learning success through a digital promise can effectively support first-year students in their educational journey. This approach helps develop time management skills and encourages responsibility for class attendance, serving as an introduction to the expectations for successful learning in higher education. Ultimately, taking responsibility and cultivating self-discipline are significant challenges that first-year university students encounter [38].

Student Research and Inquiry-based Learning

Encouraging students to engage in research activities early in their academic journey promotes inquiry, critical thinking, and a deeper understanding of subject’s matter. Inquiry-based learning allows students to explore questions, investigate problems, and conduct research projects, leading to a more profound engagement with the curriculum. Institutional support for undergraduate research programs and student-led conferences can further enhance this aspect of learning. The study by Spence et al. (2020) encourages students to "think critically and in new ways about the subject matter." The student-scientist curriculum is most effective when implemented by supportive and optimistic instructors, whose positive energy motivates students to successfully design, conduct, and communicate collaborative projects. While these research projects may not contribute new knowledge to the scientific community, they provide authentic research experiences for our student population [39].

The study by Wierzchowski et al. (2013) identified two key theoretical frameworks: meaningful learning and situated cognition. In meaningful learning, students establish clear connections among the cognitive, psychomotor, and affective domains, which enhances their motivation to engage in future research and helps them relate research to real-world problems. This highlights the importance of linking research to students' personal lives and career interests. In the context of situated cognition, learning what to do (cognitive) and how to do it (psychomotor) often stems from experiences of failure, such as obtaining negative research results, which are a natural part of the research process, even if they are rarely published in scientific journals [40].

Inclusivity and Diversity in Learning Ecosystems

A truly effective ecosystem in inclusive and respects diversity. Integrating culturally responsive pedagogy and ensuring accessibility in education allows students from diverse backgrounds to thrive. Emphasizing equity and inclusion ensures that all learners have equal opportunities for success, contributing to a more just and empathetic society [41,42]. To promote equal status among groups, establishing common goals, facilitating intergroup cooperation, and securing support from authorities are essential. Preparing students to form friendships with peers from ethnically diverse backgrounds equips them with valuable skills for future educational, community, and workplace environments. To enhance cultural diversity and support for students, schools can take several actions: hire diverse staff and create pathways for training and hiring from the local community, utilize curriculum materials that accurately reflect economically and racially diverse students and families, and employ a parent outreach coordinator to recruit a diverse student population and support families from various backgrounds [43].

Manase and Ngubane S (2024) emphasize that recognizing and addressing students' diverse needs enables supervisors to tailor their approaches effectively, ensuring that students receive the necessary support to enhance their academic progress. Supervisors should remain engaged with the challenges students face, fostering an environment that promotes their potential and aligns with the principles of humanizing pedagogy. Additionally, open and distance e-learning institutions need to implement reasonable policies and accommodations for postgraduate students to facilitate timely completion of their studies, thereby ensuring a return on investment for students, universities, and the nation. University libraries must also provide resources that meet the research needs of students with disabilities. Furthermore, laboratories should be fully accessible, and dedicated online support spaces should be established for postgraduate students with disabilities, which would also help train academics in effectively supervising these students [44]. 

The Institutional Effectiveness Partnership Initiative (IEPI), launched in 2014, aims to support districts in enhancing student success and improving their fiscal and operational effectiveness. It focuses on four key areas: (1) student performance and outcomes; (2) accreditation status; (3) fiscal viability; and (4) programmatic compliance with state and federal guidelines. The initiative emphasizes various aspects, including enrollment management, integrated planning and resource allocation, research for institutional effectiveness, governance, decision-making, and technology. Faculty participants reported implementing equitable practices in their classrooms by modifying course curriculum and improving interactions with students, such as emphasizing the importance of language, identity, and humility. The initiative also includes five overarching categories: informational webinars, in-person networking events, year-long programs, communities of practice, and a cohort-based intersegmental partnership internship program. Overall, respondents found the informational webinars to be moderately to quite useful in achieving the goals set by the vision and IEPI mandates. By addressing institutional barriers and investing in professional development, the initiative equips colleges and districts with the necessary tools to meet evolving challenges and foster a culture of innovation, ultimately providing high-quality education for students [45].

Continuous Professional Development for Educators

The role of educators is pivotal in creating effective learning ecosystems. Ongoging professional development ensures that educators are equipped with the latest pedagogical skills and knowledge. Workshops, peer observation, and innovative in their teaching approaches. Establishing a professional learning community for cultivating future design talents using a "peer coaching" mechanism [46].

Mazur et al. (2023) emphasize that a continuing professional development (CPD) system should involve attending lectures and professional seminars, as well as engaging with scientific and practical publications during free time. The need for these seminars is justified by their role in allowing professionals to reflect on their experiences and training, which fosters a deeper understanding of clinical cases. Notably, every fourth doctor reports gaining essential knowledge for professional growth by learning from colleagues, highlighting the importance of collaboration, trust, and respect in addressing complex diagnostic and treatment challenges [47].

According to Nguyen et al. (2024), the lesson study approach offers three key benefits: it facilitates teacher collaboration, enhances their understanding of lesson content and student learning processes, and boosts their motivation for continuous professional development (CPD) [48]. Additionally, Adane et al. (2024) found that participants engage in formal activities like induction programs, training, and conferences, while also benefiting from informal learning opportunities within their daily work, such as discussions, reading, and utilizing electronic resources to enhance their teaching and research skills [49].

Key Findings

In an increasingly complex educational environment, developing a learning ecosystem that is adaptive, inclusive, and prepares students for global challenges is essential. The foundation of such an ecosystem is built on evidence-based practices, which rely on research-backed teaching methods like formative assessments and spaced repetition to enhance learning outcomes. By adopting strategies proven effective through empirical research, educators can create more impactful educational experiences.

Cultivating global citizenship is another critical component, achieved by embedding global issues and cross-cultural competence within curricula. This approach fosters students’ awareness of global challenges and encourages them to act as socially responsible citizens. Integrating the United Nations’ SDGs into educational programs aligns learning objectives with larger global priorities, promoting environmental and social responsibility while inspiring students to contribute to a sustainable future. Promoting translatory practice bridges the gap between theory and application, enabling students to connect classroom knowledge with real-world contexts. Project-based learning, internships, and community engagement initiatives offer practical experiences that translate academic theories into actionable skills. Alongside this, active learning techniques like problem-based learning.

## Conclusions

In conclusion, the development of a robust learning ecosystem is critical for preparing 21st-century learners to thrive in an increasingly complex and interconnected world. By integrating strategies such as evidence-based education, global citizenship, SDG-focused learning, translatory practices, active learning methods, improved clinical placements, and peer learning, educational institutions can create environments that foster both personal and professional growth. These approaches not only enhance the effectiveness of learning but also cultivate adaptable, socially responsible, and globally aware individuals. As education continues to evolve, adopting these strategies is essential to equipping students with the skills and mindset needed for future challenges and opportunities.
